# Bringing the Reading Sciences Into the Classroom: Insights for Phonics Instruction

**DOI:** 10.1177/17456916261438004

**Published:** 2026-05-05

**Authors:** Tobias Ungerer, Kathleen Rastle, Blair C. Armstrong

**Affiliations:** 1 Department of Psychology, University of Toronto Scarborough; 2 Department of Psychology, Royal Holloway, University of London; 3 Basque Center on Cognition, Brain and Language, San Sebastián, Spain

**Keywords:** language, communication, education, literacy, reading acquisition, phonics instruction, curriculum design, classroom practice

## Abstract

Teaching phonics—that is, systematic mappings between letters and sounds—plays a foundational role in how children learn to read in alphabetical writing systems. Although the reading sciences yield important insights into the factors underlying effective phonics instruction, these findings have not been sufficiently linked to key decisions that teachers must make in the classroom—for instance, which spelling-sound regularities to teach, in what order to introduce them, how to illustrate them with example words, and when to teach exception words. We first show that existing phonics programs provide varying guidance on these aspects, which may affect learning outcomes in ways that are poorly understood. We then discuss how research on reading and learning can inform key considerations regarding the use of effective phonics content. We also highlight gaps in current knowledge that remain to be addressed by further work. Finally, we outline a road map for how future research could support the design and selection of optimized phonics content, thus benefiting the professional practice of diverse stakeholders in education.

Reading is a key determinant of individual and societal success and well-being. Better reading skills are connected to higher academic achievement (Rabiner et al., 2016), increased income (McLaughlin et al., 2014), economic growth (Goczek et al., 2021), better health (Fleary et al., 2018), and more active civic participation (Farrell et al., 2022). Yet these and other positive outcomes are jeopardized by current trends in literacy rates observed in many countries around the world. A 2021 survey of 48 countries and regions—the majority of which display high levels of income—found that, on average, two thirds of grade 4 students do not learn to read with a high level of comprehension (Mullis et al., 2023). The situation is much exacerbated in middle- and low-income countries, where an estimated 57% of children do not even reach a minimum level of reading comprehension by age 10 (The World Bank, 2019). Comparisons with previous years show that these figures have recently increased, a negative trend that is also apparent in other international reading assessments (Organisation for Economic Co-operation and Development, or OECD, 2023). In particular, the schooling disruptions caused by COVID-19 have led to substantial drops in reading performance, especially among students from disadvantaged groups (Kuhfeld et al., 2023; Relyea et al., 2023).

An important tool in addressing these challenges is provided by the vast body of research on reading and literacy education, known as the “science of reading” (e.g., Seidenberg, 2013). This work comprises, among other things, foundational theories of reading acquisition (e.g., Cain et al., 2017; Castles et al., 2018; Duke & Cartwright, 2021), comparisons of different modes of reading instruction (e.g., National Reading Panel, 2000; Suggate, 2016), and demonstrations of the influence that diverse sociocultural and individual-level cognitive factors have on literacy development (e.g., Connor et al., 2014; Durgunoglu & Verhoeven, 2013). Here, we use the plural term “reading sciences” to highlight the multidisciplinary nature of this research, which includes cognitive science, education, developmental psychology, and other fields, such as linguistics and neuroscience. There is, however, growing awareness that many key findings from reading research have been insufficiently linked to instructional practice in the classroom, leading to widespread calls for translational research that bridges this divide (Hindman et al., 2020; Petscher et al., 2020; Seidenberg et al., 2020; Shanahan, 2020; Solari et al., 2020). In Seidenberg et al.’s words (2020, p. S121), “although reading science is highly relevant to learning in the classroom setting, it does not yet speak to what to teach, when, how, and for whom at a level that is useful for teachers.” The problem, in fact, goes both ways: Neither do insights from the reading sciences sufficiently inform real-world decision-making in the classroom, nor are practitioners’ knowledge and experience routinely considered as part of the scientific process.

In this article, we aim to reduce the translational gap in an area that is central to the earliest phase of reading instruction: the use of phonics to teach systematic mappings between letters (e.g., “e”) and sounds (e.g., /ε/, as in *bed*), also called grapheme-phoneme correspondences (GPCs)—see Figure 1 for further illustration of this concept.^1^ After decades of debate about the benefits of phonics versus whole-word (or whole-language) instruction (Pearson, 2004), there is now widespread consensus that phonics provides the most effective means of teaching children how the visual symbols of writing map to spoken language in alphabetical writing systems (Ehri et al., 2001; Galuschka et al., 2014; McArthur et al., 2018; Suggate, 2016; but for critical perspectives, see Bowers, 2020, 2021). Phonics plays a foundational role at the outset of reading instruction: A small set of GPCs enables children to “decode” a very large number of written words by linking their spelling to the phonology and meaning of spoken words they already know (Solity & Vousden, 2009; Vousden, 2008). Because teaching phonics gives students the tools to read independently outside the classroom, it can trigger positive snowball effects whereby better reading skills enable additional print exposure, which in turn fosters reading fluency (Stanovich, 1986; Torppa et al., 2020; for the directionality of these relationships, see Erbeli et al., 2020; van Bergen et al., 2021). As children’s reading abilities develop, phonics-driven word decoding increasingly interacts with other skills, such as the automatic recognition of whole word forms, the self-teaching of orthographic regularities, and the application of fine-grained semantic and morphological knowledge (for an overview, see Castles et al., 2018). Phonics instruction thus plays a fundamental role in the long journey to skilled reading. This is illustrated by a large-scale study in England (McGrane et al., 2017) showing that phonics proficiency in Year 1 (ages 5 to 6) is the strongest predictor of reading comprehension 4 years later, exceeding the effects of other factors, such as student demographics, home literacy environment, and school performance.

**Fig. 1.**
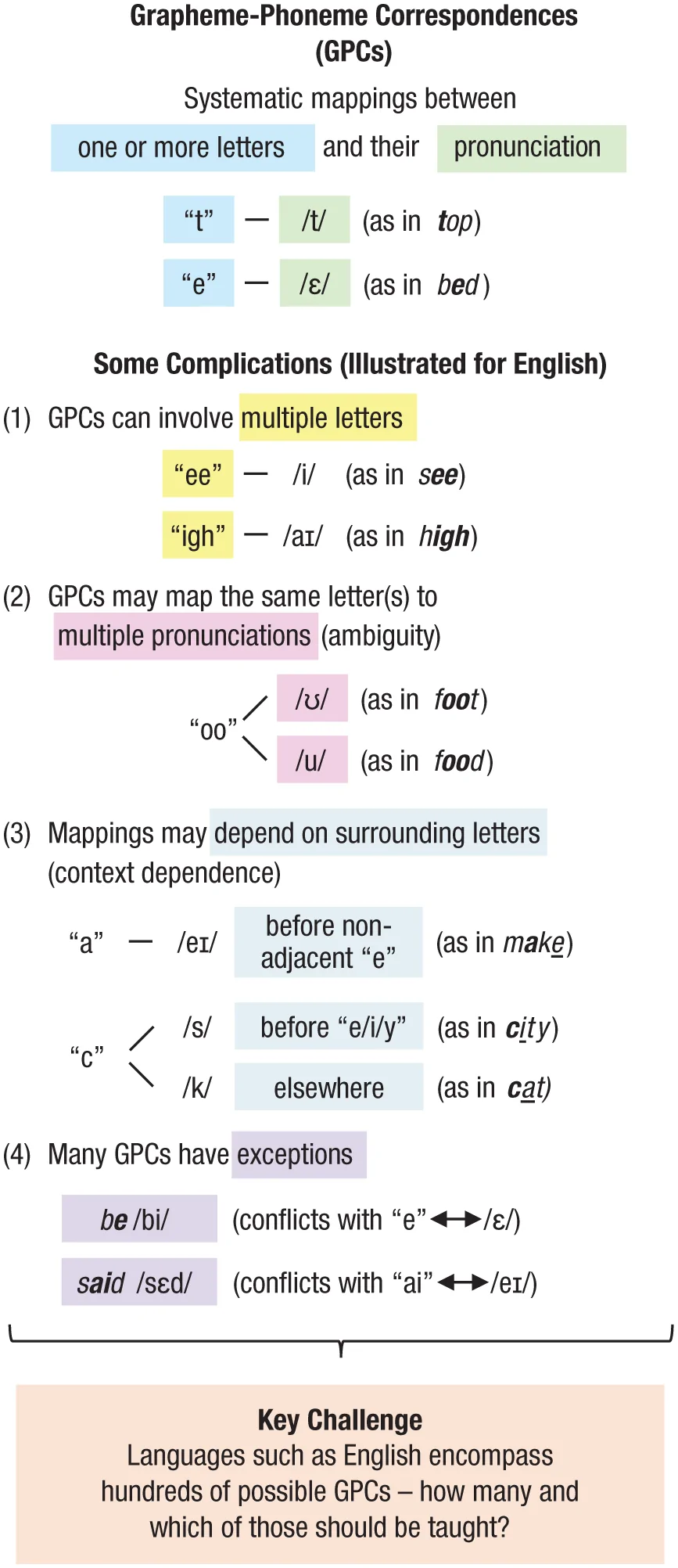
Overview of grapheme-phoneme correspondences (GPCs) and associated challenges.

However, teaching and learning about phonics poses significant challenges both for students and instructors (see Fig. 1). Throughout this article, we illustrate these issues in English because (a) it is the most widely spoken language globally, including nonnative speakers (Ethnologue, n.d.); (b) children in many countries, especially low- and middle-income countries, are taught to read in English even if it is not their native language (for discussion, see Milligan & Tikly, 2016); and (c) English displays a high degree of orthographic irregularity—that is, the same letter may be involved in the production of different sounds in different contexts, such as “e” in *mane*, *elephant*, and *team.* This irregularity makes English particularly difficult for beginning readers (Seymour et al., 2003). Nevertheless, the broader questions we address apply to many other alphabetical writing systems, even if the answers may depend on language-specific features.

One basic challenge is that it is not clear which and how many GPCs should be taught at school. Scientific studies have identified up to 500 or more potential GPCs in English (Brooks, 2015; Gontijo et al., 2003), but only a fraction of these (typically less than 100) are taught, given the classroom time allocated to reading instruction. Teaching GPCs is further complicated by the fact that they may apply to units of varying grain sizes, including single-letter graphemes (e.g., “t” – /t/, as in *top*) as well as multiletter graphemes (e.g., “ee” – /i/, as in *see*, or “igh” – /ai/, as in *high*), thus requiring learners to consider several possible segmentations of the same word. In addition, individual graphemes often have multiple pronunciations: For instance, “oo” can map to either /ʊ/ (as in *foot*) or /u/ (as in *food*). Moreover, some pronunciations are context-dependent, as is the case for “a,” which typically maps to /æ/ (as in *map*) but is pronounced as /ei/ before a nonadjacent “e” (as in *make*). This raises the question of whether these potentially ambiguous mappings should be taught and, if so, in what order.

Furthermore, effective phonics instruction requires additional components, whose importance may have been underestimated to date. For instance, GPCs need to be practiced with example words in which they occur, such as using the words *bed*, *get*, and *hen* to illustrate the mapping between “e” and /ε/. But how many example words should teachers provide per GPC? What are the characteristics of effective examples? Moreover, phonics instruction involves the teaching of exception words (or “tricky words”) that do not follow the regularities learned through GPCs, such as *be*, which is pronounced as /bi/ and thus diverges from the regular “e” – /ε/ mapping, or *said*, which is pronounced as /sεd/ and conflicts with the “ai” – /ei/ rule. This raises further questions: Which exception words should teachers introduce, and when is the right time to teach them?

In seeking to address these questions, teachers, schools, and education boards cannot rely solely on the variety of available published phonics programs (also called *schemes* or *curricula*). Even though these programs provide valuable guidance on many aspects of phonics instruction, we will show below that they differ substantially in the teaching content they specify across each of the abovementioned components (GPCs, example words, exception words; see also Solity, 2020). The methods and rationales underlying the design of these programs remain largely nontransparent, so “almost every reading curriculum can claim an equally loose connection to the ‘science of reading’” (Seidenberg et al., 2020, p. S122). This makes it difficult for researchers, teachers, schools, and educational authorities to identify an optimal set of phonics content that can be used to maximize students’ learning outcomes.

How can the reading sciences help? As Seidenberg et al. note (2020, p. S122), phonics instruction is marked by an “absence of sufficient translational research,” given that “the research literature does not provide detailed guidance about which spelling-sound patterns to teach; how many to teach; whether patterns should be taught in isolation [. . .] or in disambiguating contexts [. . .]; or other issues that have to be adjudicated for instruction to proceed.” In this article, we strive to narrow this gap in two ways. First, we identify key aspects of phonics knowledge that differ among published programs and discuss how empirical findings and methodological tools from reading research can inform the selection of effective phonics content in these areas. Even in cases in which specific aspects of a program have, to date, not been addressed by the reading sciences per se, we show that insights from other areas of learning research, such as category learning and language acquisition more generally, can be fruitfully transferred to phonics instruction. In doing so, we situate our approach in a long tradition of connecting research on the psychology of learning with practical issues in the classroom, as advocated, for instance, by the paradigm of instructional design (Simmons & Kameenui, 1998; Sweller et al., 1998). Second, we highlight open questions with respect to phonics content that could benefit from additional targeted research. Putting these two first parts together, we then outline a road map of what we know and what future researchers should tackle next to support teachers and other stakeholders in education in designing and selecting effective phonics content.

## Teaching GPCs: Which and How Many?

GPCs lie at the heart of phonics instruction: They represent the core knowledge that students must acquire to map letters to sounds. A first and fundamental set of decisions that instructors must therefore make is which and how many GPCs to teach. Existing phonics programs provide a range of different answers to these questions. Here and in subsequent parts of this article, we illustrate this with a recent analysis (Rognan, Champagne, et al., 2026) of three widely used programs: (a) Jolly Phonics (Lloyd & Wernham, 2014), a long-standing commercial program first published in 1992 that has been used in over 150 countries, according to its publisher (Jolly Learning, n.d.); (b) Letters and Sounds, introduced by the United Kingdom’s Department for Education and Skills (2007a) when systematic phonics instruction was mandated in England, where it subsequently became the most popular program (Hodgson et al., 2018); and (c) Read Write Inc. (Miskin, 2020), a more recent commercial program used in the United Kingdom, the United States, Australia, the Middle East, Europe, and Asia, according to the publisher (Ruth Miskin Training, n.d.). All three programs have been used in thousands of schools to teach millions of students, and they thus illustrate major trends in current English-language phonics instruction.

### Differences in GPCs taught

**Fig. 2.**
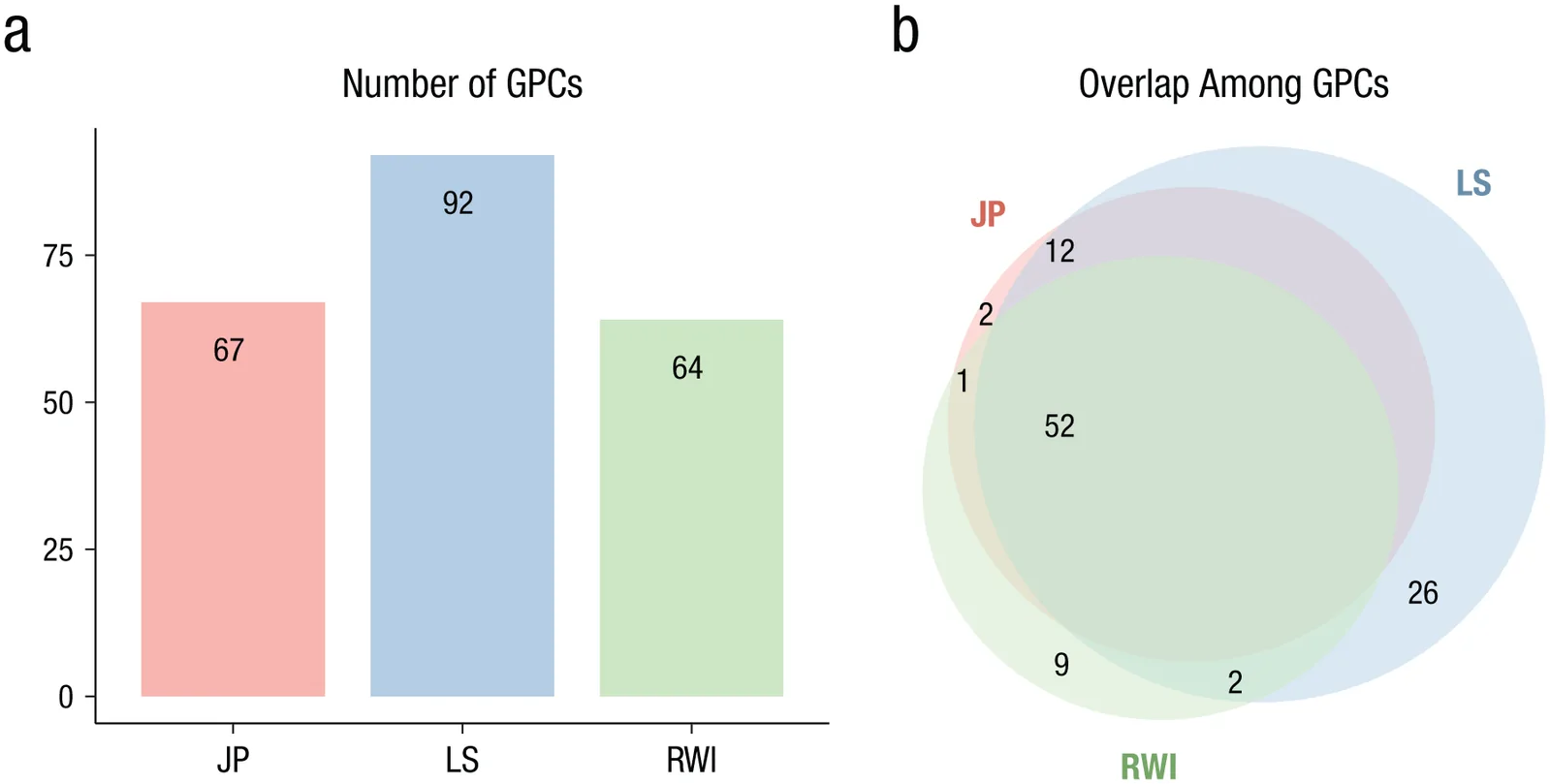
Comparison of (a) the number of grapheme-phoneme correspondences (GPCs) and (b) overlap among GPCs taught in three widely used phonics programs. JP = Jolly Phonics; LS = Letters and Sounds; RWI = Read Write Inc. From Rognan, Champagne, et al. (2026). CC BY 4.0.

Figure 2 provides a summary comparison of the GPCs introduced by each program (based on hand-coded data from Rognan, Champagne, et al., 2026).^2^ As Figure 2a shows, the programs vary in the total number of GPCs they teach: Jolly Phonics and Read Write Inc. introduce similar numbers (67 and 64 GPCs, respectively), whereas Letters and Sounds teaches 92 GPCs, which is approximately 40% more than the other two programs. Moreover, as illustrated in Figure 2b, there is only partial overlap among the specific GPCs taught. In this diagram, each program is represented by a colored circle; the size of the circles corresponds to the number of GPCs taught; and the intersections of the circles illustrate how many GPCs are identical across two, or all three, programs. This analysis reveals a common core of 52 GPCs used across all three programs. However, it also shows that some GPCs are used by only two of the three programs, and that each program contains a number of unique GPCs that are not shared with either of the other programs. As might be expected given its total GPC count, Letters and Sounds contains the largest number of such unique GPC: For instance, it teaches four distinct pronunciations for the letter sequence “ou” (/aʊ/ as in *out*, /oʊ/ as in *mould* [e.g., in the Canadian spelling], /ʊ/ as in *could*, and /u/ as in *soup*), whereas the other two programs introduce only the most frequent “ou” – /aʊ/ mapping. However, even Read Write Inc., despite teaching the smallest set of GPCs overall, includes nine GPCs (14% of its total set) that are not shared with the other programs, such as “ire” – /aiɹ/ (as in *fire*) and “tion” – /ʃən/ (as in *station*).^3^

Considering the central role of GPCs in phonics instruction, it is possible that the substantial variability among GPCs used by popular phonics programs leads to differences in their efficacy, all other factors being equal. For example, a given program may outperform others because it teaches more GPCs overall, because it introduces a more effective set of GPCs, or because it selects an appropriate number of the most relevant GPCs and thus gets the best “bang for the buck.” However, in the absence of detailed information on the publishers’ part, the way in which existing programs design their GPCs remains a mystery. In particular, it is unclear what considerations (if any) from the reading sciences have influenced these decisions. This leads us, next, to discuss how reading research can provide evidence-based guidance on the factors and considerations underlying the choice of GPCs.

### Three insights from reading research

A first way in which the reading sciences can inform the selection of appropriate GPCs is by shedding light on how the properties of orthographies determine the set of phonics regularities that readers must acquire in their respective language. English, for instance, is an orthographically deep language (Katz & Frost, 1992; Schmalz et al., 2015; Ziegler & Goswami, 2005): That is, it displays many complex regularities, which involve not only one-to-one mappings between single letters and sounds but also more complex mappings, such as multiple letters mapping to a sound—for example, “ee” corresponding to /i/ (as in *see*), or mappings that apply only when a letter occurs in a certain context, such as “a” being pronounced as /ei/ when followed by a nonadjacent “e” (as in *make*; see Fig. 1 for further examples). In addition, there are substantial inconsistencies among regularities, with different pronunciations existing for the same letter sequence. As noted above, the sequence “ou,” for example, can be pronounced in several ways, including /aʊ/ (as in *out*), /oʊ/ (as in *mould*, e.g., in the Canadian spelling), /ʊ/ (as in *could*), and /u/ (as in *soup*).

Teaching even just the more frequent of these complex and inconsistent mappings would require upward of 170 GPCs (Rastle & Coltheart, 1999; Vousden, 2008). This is roughly twice the number (or more) of what existing programs teach, and it would require a dramatic increase in instructional time focused on reading skills, with classroom time already being a very limited resource. Furthermore, investing more time into teaching GPCs delays the gradual transition from isolated word practice to text reading that typically occurs during the later stages of phonics instruction after a substantial number of GPCs have been taught. This delay may impede students’ progress in acquiring other reading-relevant knowledge and skills, such as reading fluency (i.e., the speed and accuracy of reading), vocabulary development, and reading comprehension (i.e., the ability to understand what is read; Cunningham & Stanovich, 1991; Spear-Swerling, 2006; Torppa et al., 2020).

Second, and relatedly, reading research provides a rationale for why phonics instructors may teach only a subset of GPCs explicitly, whereas further spelling-sound regularities can be learned implicitly through exposure to text. On the one hand, there is strong evidence that explicit rule teaching—that is, presenting students with GPCs and practicing them by reading aloud example words in which the GPCs occur—significantly boosts reading acquisition (Bitan & Booth, 2012; Kirschner et al., 2006; Rastle et al., 2021). A recent meta-analysis (Ren et al., 2024) found a large positive effect of explicit instruction on phonics acquisition across more than a dozen experimental studies.

On the other hand, lab experiments have demonstrated that children and adults can also acquire spelling-sound mappings implicitly by extracting statistical regularities from the text input that they are exposed to. For example, studies have found that students from first grade onward are sensitive to orthographic regularities that are typically not taught explicitly, such as constraints on the use of double consonants (Pacton et al., 2001) or the ways in which vowel pronunciations are influenced by neighboring consonants (Treiman et al., 2003, 2006; see also Arciuli & Simpson, 2012, for general effects of statistical-learning ability on reading performance). However, such implicit learning typically progresses quite slowly (Steacy et al., 2019; Treiman et al., 2006), may only yield incomplete acquisition of complex spelling-sound regularities (Treiman & Kessler, 2019; Treiman et al., 2003), and may leave some readers behind (Rastle et al., 2021). Additional evidence for implicit learning comes from computational simulations of reading that are based on key principles of how the human brain learns, represents, and processes information (Harm & Seidenberg, 2004; Perry et al., 2007; Plaut et al., 1996; Seidenberg & McClelland, 1989). These models are able to infer spelling-sound regularities from large sets of example words, based solely on their ability to segment letters and sounds and associate them with each other, without any explicit knowledge of GPCs. Given these observations, a central task in phonics instruction is to decide which GPCs to teach explicitly and which ones to leave for students to acquire implicitly.

A third contribution of the reading sciences has been to identify factors that may determine this trade-off between explicit instruction and implicit learning. One viable strategy may be to teach only the most frequent GPCs explicitly (Solity, 2020; Solity & Vousden, 2009). Prior work has revealed stark differences in the frequency with which GPCs are used: Whereas a few GPCs apply to a large number of words, many other GPCs are only rarely used, with the overall frequency distribution approximately following an exponential curve (Vousden, 2008). Teaching many infrequent GPCs explicitly would thus provide diminishing returns and may not reflect the best overall use of instructional time. Another factor to consider is that, as noted above, GPCs vary in their consistency: For instance, “ee” is almost always pronounced as /i/, whereas “ou” has many alternative pronunciations (Fry, 2004). Other GPCs create further inconsistencies by specifying pronunciations for a letter sequence, such as “oe” – /oʊ/ (as in *toe*), which override previously acquired rules for the individual letters, such as “o” – /a/ (as in *top*) and “e” – /ε/ (as in *bed*). The question is how many such alternative or overlapping pronunciations, even if they are frequent, should be taught, given that they may create cases of ambiguity in which children are no longer confident which GPC to apply.

To quantify these relationships, Rognan, Ungerer, et al. (2026) used a computational algorithm to generate all possible pronunciations of a set of words from naturalistic texts read by children, given the GPCs taught in different phonics programs. They found that programs that teach more, including inconsistent, GPCs increase the proportion of words that can be theoretically decoded. That is, among all the GPCs that apply to a given word, a specific (sub)set of rules produces the correct pronunciation, even if other GPCs produce alternative, incorrect pronunciations. At the same time, programs that teach fewer GPCs increase the proportion of words that can be uniquely decoded—that is, where only a single set of GPCs applies to the letters of a word, and this set yields the correct pronunciation. However, it is unclear which of these two dimensions—the potential ability to read a word, or the certainty of choosing the correct pronunciation among multiple possible options—should be emphasized at the outset of literacy education. Considering the opposing pressures at hand, we expect that effective phonics instruction involves a trade-off between the two, which may be determined both by the frequency and the consistency of GPCs.

### Interim summary and future directions

In sum, the reading sciences provide a theoretical and empirical framework for addressing the question of which and how many GPCs to teach. In particular, prior work has shown how a combination of explicit instruction and implicit learning can be used to acquire complex phonics knowledge in languages such as English, and what factors may constrain how GPCs are selected for explicit teaching. However, existing work provides little concrete guidance on what an optimal GPC set should look like; although some suggestions have been made (e.g., Solity, 2020), their effects on reading acquisition have not been systematically assessed.

Future research could draw on a variety of empirical methods to compare the efficacy of existing GPC sets and design optimized GPC content. For example, controlled lab experiments could be used to study how the number, frequency, and consistency of explicitly taught GPCs affect reading outcomes. The findings may inform the development of optimized GPC sets that can then be confirmed in ecologically valid settings using in-class intervention studies. At the same time, computational models of skilled reading and reading acquisition (Coltheart et al., 2001; Perry et al., 2007; Plaut et al., 1996) could be used as a resource-efficient alternative to studies with human participants. Following prior demonstrations that explicit GPC teaching can be integrated into these models (Hutzler et al., 2004; Powell et al., 2006), future research could use them to compare how the varying GPC sets used by phonics programs influence learning and to support the optimized selection of GPCs (see Armstrong et al., 2024, for an illustration of this approach).

## The Order in Which GPCs Are Taught: Not Just a Matter of Complexity

Apart from deciding which GPCs to teach, another important set of decisions that phonics teachers must make concerns the order in which to introduce GPCs. A range of factors could determine an effective teaching order for GPCs. For instance, one commonly noted strategy (Roembke et al., 2020; Vadasy & Sanders, 2021) is to introduce single-letter GPCs, such as “e” – /ε/ (as in *bed*), before more complex GPCs that may either involve multiple letters, such as “ee” – /i/ (as in *see*), or that may be context dependent, such as “a” – /ei/ before nonadjacent “e” (as in *make*). A relevant question is to what extent different phonics programs adopt this simple-before-complex strategy.

### Differences in GPC teaching order

**Fig. 3.**
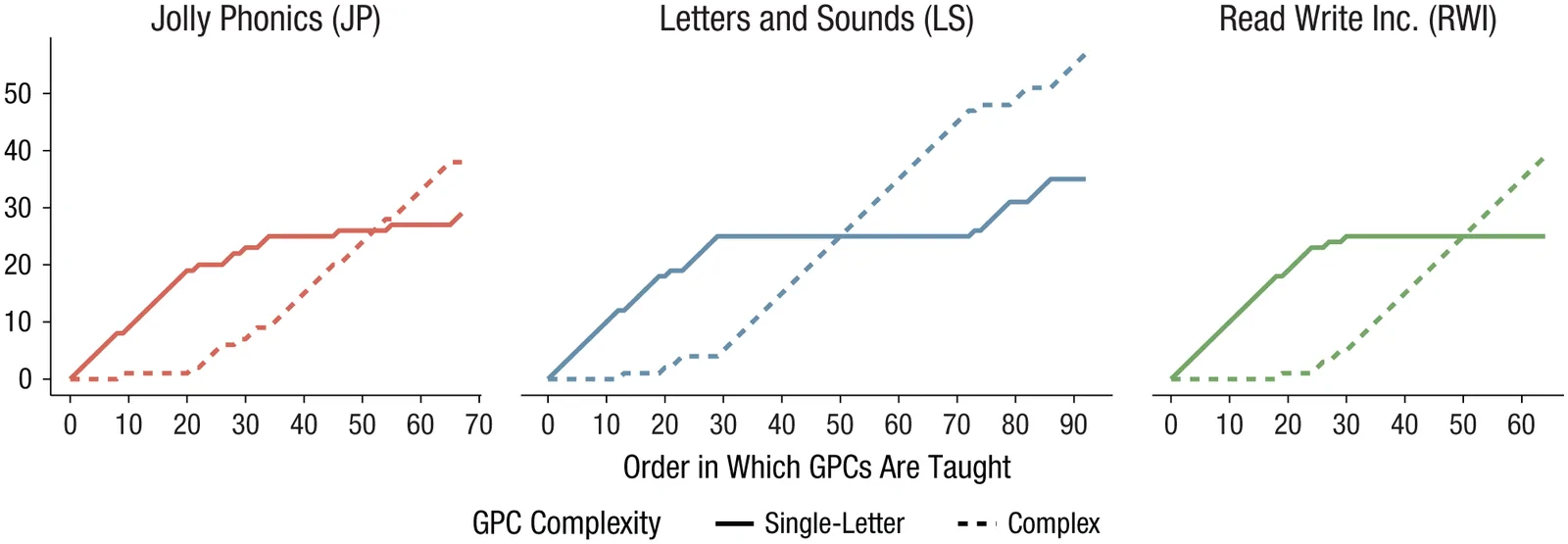
Cumulative number of single-letter and complex grapheme-phoneme correspondences (GPCs) taught in three phonics programs. From Rognan, Champagne, et al. (2026). CC BY 4.0.

Figure 3 breaks down the order in which single-letter and complex GPCs are introduced in the three phonics programs described earlier (based on Rognan, Champagne, et al., 2026).

On a coarse level, all three programs appear to follow a similar strategy, with most single-letter GPCs being taught early on and most complex GPCs being introduced later. Nevertheless, the diagrams also reveal finer-grained differences between programs. Read Write Inc. is the strictest adopter of the simple-before-complex strategy: As shown on the right of Figure 3, the first 25 GPCs are almost all single-letter, whereas the remaining GPCs taught after that are primarily complex. Jolly Phonics (at left) and Letters and Sounds (center), on the other hand, both introduce single-letter GPCs in parallel with some complex GPCs in the early stages of the programs (e.g., “ck” *–* /k/, as in *pick*, or “ai” – /ei/, as in *aim*). Moreover, both programs teach additional single-letter GPCs later on in the programs, many of which specify alternative pronunciations of previously taught letters (e.g., “y” – /i/, as in *happy*, and “y” – /ai/, as in *dry*, both of which conflict with “y” – /j/, as in *year*).

### Complexity, consistency, and other factors

Given the variation in GPC teaching order among programs, reading research may be able to shed light on the benefits of teaching simple before complex GPCs. Indeed, there are several arguments that speak against a strict adoption of the simple-before-complex strategy. First, some multiletter GPCs, such as “sh” – /ʃ/ and “ee” – /i/, are not only frequent but also highly consistent (Fry, 2004)—that is, they apply to the vast majority of words that contain the respective letter sequence (with few exceptions, such as the pronunciation of *been* with short /i/ in some varieties of English, where it rhymes with *tin*). In contrast, some single-letter GPCs are inconsistent, including most vowels but also consonants such as “c,” which can either be pronounced as /k/ (*cat*) or /s/ (*city*), depending on the following letters. Teaching frequent and consistent multiletter rules earlier may therefore provide students with more reliable cues for reading than teaching less frequent or less consistent single-letter rules (see Dehaene, 2010, Chapter 5, for a discussion). This is also in line with evidence that kindergartners with minimal formal phonics education are more likely to pronounce graphemes correctly if they map to a single phoneme or do not share their pronunciation with other graphemes (Huang et al., 2014). Similarly, children up to grade 3 perform better on pronouncing frequent and consistent graphemes (Larsen et al., 2020; Sprenger-Charolles et al., 1998). The fact that infrequent and inconsistent GPCs are more difficult to learn suggests, at least tentatively, that they should be taught later, even though future work is needed to explicitly compare how different GPC teaching orders affect reading outcomes.

Another argument against the simple-before-complex strategy is that even though some complex GPCs, especially context-dependent rules, may be difficult to acquire (Larsen et al., 2020; Samara & Caravolas, 2014; Treiman & Kessler, 2019), other complex GPCs are not necessarily more challenging for learners than single-letter GPCs. Vadasy and Sanders (2021, Study 2) found that kindergartners (who had little prior phonics knowledge) were able to learn single-letter and two-letter GPCs equally well across a range of reading and spelling tasks. Further evidence from Roembke et al. (2020) suggests that even teaching single-letter GPCs, such as “e” – /ε/ (as in *bed*), alongside multiletter GPCs that link the same letter to a different pronunciation, such as “ea” – /i/ (as in *sea*), which also contains the letter “e,” does not impede learning. Roembke et al. found that first-grade students acquired such “overlapping” GPCs (i.e., that share letters) better than “nonoverlapping” GPCs (i.e., that contain only distinct letters). This suggests that new GPCs—including multiletter rules—that are similar to learners’ already existing knowledge are easier to acquire and do not lead to confusion.

Apart from complexity, the order in which GPCs are taught may be guided by additional factors. For example, as noted by Roembke et al. (2020, pp. 3–4), programs typically group rules together in sets of similar speech sounds—for example, introducing short vowels before long vowels. Another principle that is, for example, explicitly pursued in Jolly Phonics (Lloyd & Wernham, 2010, p. 9), is to teach similar letters or sounds (e.g., “b” and “d”) several days apart to help students discriminate between them. However, the respective benefits of these strategies have not been sufficiently examined in prior research.

### The microsequencing of GPCs

Other issues that may be informed by the literature on reading and learning concern the microsequencing of GPCs—for instance, whether individual GPCs should be taught and practiced in separate blocks, or whether multiple GPCs should be introduced in parallel. General learning theory (Carvalho & Goldstone, 2015, 2017) suggests that the former strategy of *blocked learning* helps learners recognize similarities among instances of the same regularity (e.g., a single GPC), whereas the latter strategy of *interleaved learning* is beneficial for recognizing contrasting characteristics of different regularities (e.g., when discriminating between GPCs).^4^ However, most research on blocking versus interleaving to date has focused on nonlinguistic domains (for a meta-analysis, see Brunmair & Richter, 2019), with little work addressing the two learning types in the context of phonics instruction specifically (Richter et al., 2022). One exception is McMurray et al.’s (2019) study, in which first-grade students practiced GPCs in either a blocked fashion, with two new GPCs introduced in each block, or in an interleaved fashion, with six GPCs taught in parallel. No clear difference in test performance emerged between the two learning conditions. However, given that even children in the blocked condition were trained on multiple GPCs simultaneously, whereas the standard classroom practice is to introduce them sequentially at a rate of roughly one GPC per day (Lloyd & Wernham, 2010, p. 6), future work is needed to investigate blocked and interleaved learning in ecologically valid scenarios.

### Interim summary and future directions

We have illustrated that the reading sciences can shed light on how properties of GPCs, such as their complexity, influence the order in which these regularities should be taught. Future work could investigate how complexity interacts with other factors, such as the consistency of GPCs or the relative similarity between consecutive GPCs, or how key phonemic properties may affect learnability (e.g., whether a sound can be pronounced continuously, as in /s/, or only plosively, as in /p/). Further open questions concern the microsequencing of teaching, including the advantages of blocked and interleaved learning, which have not been sufficiently examined in the context of phonics instruction. Future work could draw on experimental and computational methods similar to those discussed above to investigate how multiple ordering principles affect phonics acquisition and to derive recommendations for an optimized teaching order of GPCs.

## The Use of Example Words: Diversity is Key

Discussions of phonics knowledge usually focus on the role of GPCs as the distinctive element that differentiates phonics from whole-word-based approaches and distinguishes different phonics programs from each other (e.g., Solity, 2020). What is, however, often overlooked in this context is that GPCs such as “e” – /ε/ are not taught in isolation: Rather, they are illustrated and practiced with example words, such as *bed*, *get*, and *hen*. This raises additional questions for teachers: What examples should be used in phonics instruction, and how do their properties affect learning outcomes?

### Differences in the use of example words

**Fig. 4.**
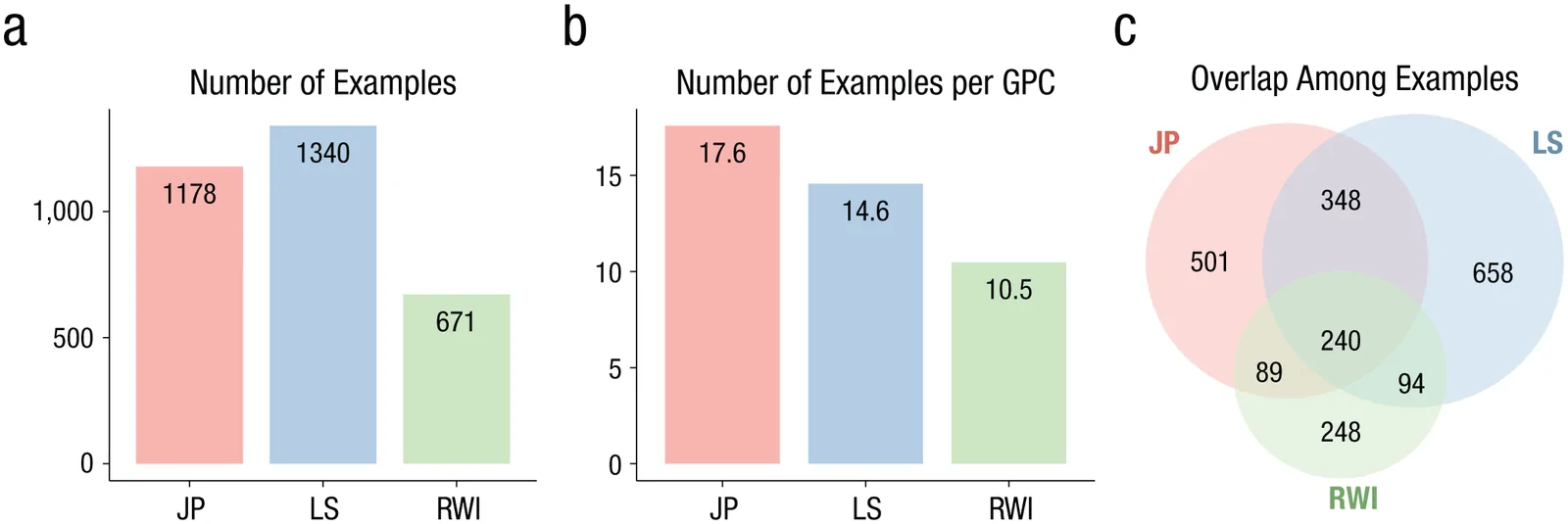
Comparison of (a) the number of example words, (b) the number of example words per grapheme-phoneme correspondence (GPC), and (c) overlap among example words taught in three phonics programs. JP = Jolly Phonics; LS = Letters and Sounds; RWI = Read Write Inc. From Rognan, Champagne, et al. (2026). CC BY 4.0.

As before, an analysis of existing phonics programs reveals substantial variability in the example words they contain. Here, we draw on Rognan, Champagne, et al.’s (2026) hand-coded lists of the curated example words that are listed in the core manuals of the three aforementioned programs. We use these as an approximation of the example words teachers may use during early phonics instruction, even though students will subsequently encounter further examples as part of decodable readers and other instruction. As shown in Figure 4a, the programs differ starkly in their total number of (distinct) examples: Letters and Sounds contains the largest set of examples (1,340 words), which is more than twice as much as for Read Write Inc. (671 words), with Jolly Phonics falling in between the other two (1,178 words).

Given that the three programs differ in their overall number of GPCs, it is also useful to compare how many examples are, on average, used per GPC. As shown in Figure 4b, Jolly Phonics provides the largest number of examples per GPC, Letters and Sounds ranks second, whereas Read Write Inc. provides considerably fewer examples per GPC. Finally, Figure 4c shows the amount of overlap among the specific example sets used by the three programs, which turns out to be rather limited. Only a minority of example words are shared across all three programs; between 37% and 49% of examples in each program are unique to that program and not shared with either of the others.

### Characteristics of effective examples

How may the aforementioned differences in the relative number and specific identity of example words influence phonics acquisition? Research studying how knowledge is learned in a broad set of domains provides extensive evidence that more diverse sets of examples lead to better acqusition of regularities (for a review, see Raviv et al., 2022). Infants, for instance, learn to discriminate between similar speech sounds better if they are pronounced by varying speakers rather than by a single speaker, suggesting that the diversity inherent in multiple pronunciations aids the formation of phonemic categories (Rost & McMurray, 2009). Similarly, both children and adults learn new words better when they encounter them in more varied sentence or discourse contexts (Hills et al., 2010; Norman et al., 2023; Rosa et al., 2017). There is also evidence that novel morphological suffixes are acquired better when these suffixes are combined with many different word stems during training (Tamminen et al., 2015). With respect to phonics acquisition, a computational simulation (Miller et al., 2020) and an experimental study (Champagne et al., 2023) provide evidence that multiple examples benefit the acquisition of spelling-sound regularities akin to GPCs. The common explanation for these results is that witnessing a recurring pattern in diverse contexts helps learners isolate that pattern, store it as a mental category that is (at least somewhat) invariant to the context in which the pattern was encountered, and then generalize it to new instances (Kemp & Tenenbaum, 2009).

Using a larger set of example words is a simple way of increasing their diversity. However, considering the instructional time needed to practice with examples, a potentially more efficient approach is to select words that are maximally distinct from each other. For instance, to practice the “e” – /ε/ mapping, teachers could use example words in which the critical vowel is surrounded by a varied set of consonants, as in *bed*, *get*, and *hen*. Experimental work by Apfelbaum et al. (2013) suggests that this increased diversity among the contextual frames in which a GPC occurs boosts learning. In their study, first-grade students practiced a set of vowel GPCs (e.g., “a” – /æ/) with example words in which the vowel was either surrounded by highly variable consonants (e.g., *pat*, *fan*, *cab*) or by consonants with low variability (e.g., *bat*, *pat*, *hat*). The high-variability condition led to significantly better learning. Subsequent research (McMurray et al., 2019; Roembke et al., 2020) has found that this effect may be modulated by additional factors, such as the difference between blocked and interleaved teaching (see “The Order in Which GPCs Are Taught” above).

Other characteristics of example words may be manipulated to further increase their effectiveness. One plausible strategy is to illustrate the same GPC in different word positions, for example by practicing the letter “p” in word-initial (e.g., *pin*), word-medial (e.g., *spy*), and word-final position (e.g., *top*). Previous research on spelling (Bernstein & Treiman, 2001) has suggested that learners encode GPCs, to some extent, in position-specific ways, thus allowing children to spell sounds more accurately when they occur in the same word position in which they have been taught. Illustrating the same GPC in varied positions should therefore help students generalize its use, while additionally exploiting the fact that letters are more easily recognizable at the word periphery, especially in word-initial position (Tydgat & Grainger, 2009), and that word-initial presentation has been shown to facilitate children’s reading and spelling acquisition (Bowman & Treiman, 2002; Treiman et al., 1993). Another possible strategy is to select pairs of contrastive example words in order to juxtapose GPCs with similar letters or sounds (e.g., *moon* vs. *noon*) or inconsistent GPCs (e.g., the pronunciation of “i” in *give* vs. *hive*). However, the validity of such a contrastive approach remains to be assessed relative to the potential benefits of interleaved learning.

### Interim summary and future directions

Together, these considerations suggest that phonics instructors should illustrate GPCs with a sufficiently large number of example words as well as examples with high word-internal variability. However, prior research has not provided concrete illustration of example sets that would fulfil these criteria. This creates tangible opportunties for future work, which could, for instance, compare example words in terms of their orthographic distinctness (Yarkoni et al., 2008) and use this measure to select examples with high word-internal variability. To decide on an optimal number of examples, we suggest that a combination of experimental work and computational modeling be used to determine an effective trade-off between the learning benefit offered by larger example sets and the increase in instructional time required to teach them.

Inspiration for this line of work could come from long-standing research on skills acquisition, which has shown that learning improvements become ever smaller as additional practice is provided, following an exponential or power-law function (Anderson, 1982; Heathcote et al., 2000). The same conclusion is also supported by large computational models of language, such as OpenAI’s GPT (Radford et al., 2019), which display important similarities with human learners, even if they do not provide complete models of human cognition. These models, too, make increasingly smaller learning gains as more training examples are provided, thus reinforcing the idea of diminishing returns (Kaplan et al., 2020). It would be fruitful to see how these learning principles play out in the context of phonics instruction, and how they affect the optimal selection of example words.

## Teaching Exception Words: A Question of Timing

As with almost any regularity in science or everyday life, there are exceptions to the rules. For example, in the context of GPCs, *be* (pronounced as /bi/) deviates from the dominant pronunciation of “e” as /ε/ (as in *bed*), and *said* (pronounced as /sεd/) diverges from the typical “ai” – /ei/ mapping (as in *rain*). Children are typically taught to recognize these exception words (also called “tricky words”) by sight—that is, via their whole-word forms—because they cannot be read accurately using the GPCs that students have been taught. The teaching of exception words is an accepted component of phonics instruction (Castles et al., 2018, pp. 14–15). Despite the shared terminology around learning these exception words by sight, this practice is unrelated to the teaching of high-frequency “sight words,” such as those on the Dolch List (Dolch, 1936) in whole-language and balanced-literacy approaches. There is substantial evidence cautioning against use of this latter practice in reading instruction (Deauvieau & Gioia, 2024; Miles et al., 2018). However, despite the widespread acceptance that exception words must be learned as whole forms in phonics programs, this practice raises a number of questions that have so far received little attention, starting with the fundamental issues of which exception words to teach and when to introduce them.

### Differences in exception words taught

**Fig. 5.**
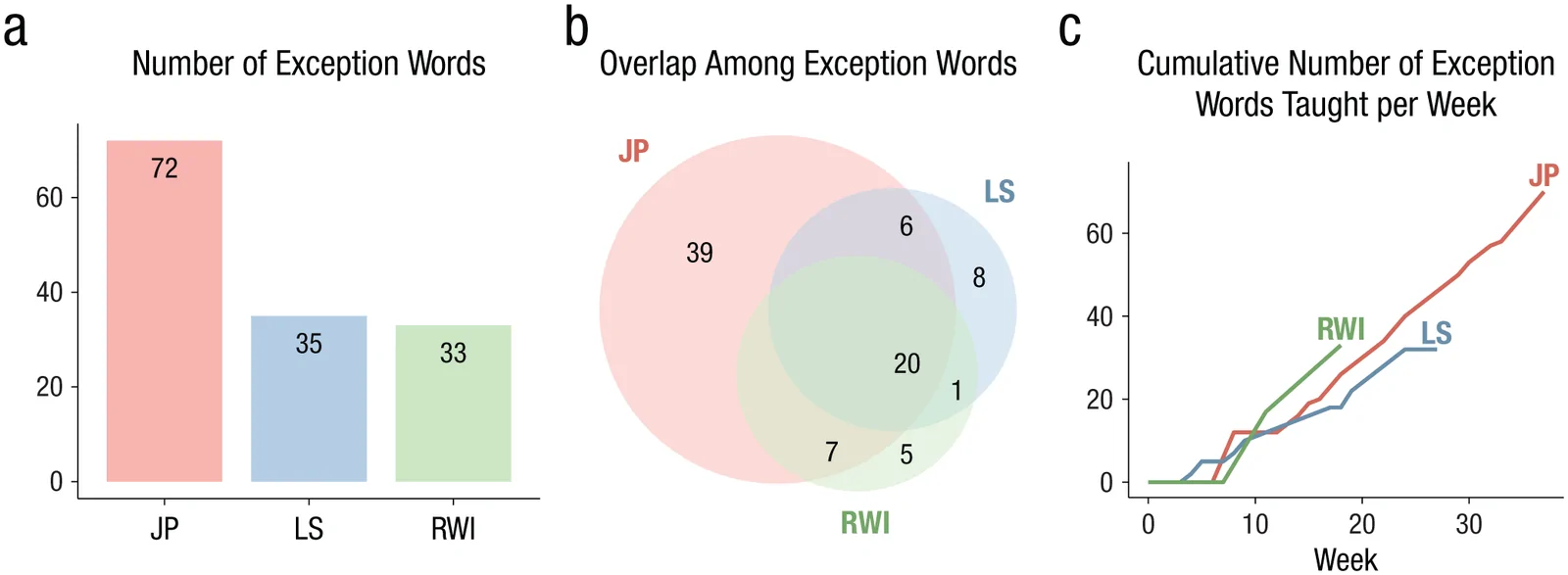
Comparison of (a) the number of exception words, (b) the overlap among exception words, and (c) the cumulative number of exception words taught per week in three phonics programs. JP = Jolly Phonics; LS = Letters and Sounds; RWI = Read Write Inc. From Rognan, Champagne, et al. (2026). CC BY 4.0.

Figure 5 provides a summary of how exception words are incorporated in the three phonics programs (based on Rognan, Champagne, et al., 2026). As Figure 5a shows, Jolly Phonics introduces around twice as many exception words as Letters and Sounds and Read Write Inc., whereas the latter two contain similar numbers of exception words. Figure 5b suggests that there is only partial overlap among the three sets of exception words: Although 20 exception words are shared by all three programs, each program contains a number of exception words (between 15% and 54% of their total set) that are not shared with either of the other programs. Finally, Figure 5c illustrates when exception words are introduced during the weekly schedule specified by the programs. Although all programs start teaching exception words fairly early, some variation is also apparent. Specifically, Letters and Sounds is the first to teach exception words, starting in Week 4 (out of 27 weeks), whereas Jolly Phonics starts a little later (in Week 7 out of 37), and Read Write Inc. is the last to teach exception words (from Week 8 out of 18).^5^

### Exceptions: a challenge for learning

Exceptions are challenging to learn because they conflict with the regularities that learners have already acquired or are in the process of acquiring. According to theories and computational models of category learning (Ashby et al., 1998; Erickson & Kruschke, 1998; Love et al., 2004; Nosofsky et al., 1994), exceptions are learned and remembered differently from regular examples, being encoded in separate memory representations that capture their atypical “special” characteristics. This is supported by neurobiological studies showing that exceptions activate specialized representations in the brain (Davis et al., 2012; Schlichting et al., 2021). As a result, exceptions are more difficult to learn than regular examples (Davis et al., 2012; Heffernan et al., 2021), even though, once learned, they are easier to recognize (Palmeri & Nosofsky, 1995; Sakamoto & Love, 2006). Applied to phonics, the acquisition of irregular pronunciations for exception words has been simulated with computational models (Kim et al., 2013; Miller et al., 2020). Their results suggest that exception words lead to small distortions in learners’ memory space, which ensure that exceptional information is kept distinct from and not confused with the information from regular examples.

The challenges associated with learning exception words should, all else being equal, encourage phonics teachers to keep their number to a minimum, especially considering the additional classroom time required to teach them. However, exception words are critical in naturalistic, child-directed reading materials: 15% to 20% of English words that are typically part of children’s oral vocabulary at the outset of literacy acquisition are not spelled in accordance with GPCs (Bowers & Bowers, 2018; Rastle & Taylor, 2018). This proportion is even higher among frequent words: Around half of the 100 most frequent English words are not pronounceable based on GPCs alone (Masterson et al., 2010; Solity & Vousden, 2009). In general, frequent words tend to be more irregular than infrequent words, presumably because infrequent irregular words would be more difficult to learn and easier to forget (Lieberman et al., 2007).

Of course, it is worth noting that even irregular words are only partly exceptional: Typically, only about a third of the sounds in exception words (e.g., the vowel in *some*) deviate from regular spelling, whereas the remaining two thirds (e.g., the consonants in *some*) follow GPCs (Rastle & Taylor, 2018). Thus, teaching a certain number of exception words as part of phonics instruction is unavoidable in preparing children to read words in naturalistic, child-directed texts. Teachers must strike a balance between minimizing the classroom time required to learn exception words—digressions that could disrupt the learning of regularities—while at the same time providing children with a base reading vocabulary needed when reading natural texts.

Another factor to consider when teaching exception words is that there is actually no clear-cut division between exceptions and GPC regularities; instead, they form part of a graded continuum, also known as a *quasiregular domain* (Armstrong et al., 2017; Seidenberg & McClelland, 1989). Some apparently exceptional words display subregularities of their own: For instance, *he*, *she*, *me*, *we*, and *be* share the same vowel pronunciation /i/, which conflicts with the dominant pronunciation of “e” as /ε/. This subregularity could alternatively be taught as a low-frequency GPC, as is the case in Letters and Sounds, where the aforementioned words are introduced as exception words in the main program but used as examples of an additional “e” – /i/ rule in supplementary material (Department for Education and Skills, 2007b).^6^ This illustrates that the decision about which words to teach as exceptions is inherently dependent on what GPCs are included in a given program and potentially the order in which subregular spelling-sound mappings are introduced (see the earlier comments in “The Order in Which GPCs Are Taught”).

### The timing of teaching exceptions

Once the set of exception words in the context of a specific program is identified, another important question is when these exceptions should be introduced. As we showed above (see Fig. 5c), the three phonics programs examined here all start teaching exception words relatively early on, even if they differ in the exact timing. Research on general category acquisition, however, suggests that later introduction of exceptions is more beneficial for learning (Heffernan et al., 2021; Mathy & Feldman, 2009). For instance, Heffernan et al. (2021) conducted an experiment in which adults learned to categorize images of flowers; apart from regular category members, exceptions were presented either from the start of the learning phase or at a later time point. Later introduction of exceptions resulted in significantly better category learning as well as better recognition of the exceptions. A plausible explanation for this is that learners first need to develop a firm grasp of the regularities before they can recognize and encode exceptions as distinct from this regular knowledge. In fact, if exceptions are taught from the start, it is possible that learners initially treat them as a general rule and then need to revise this inference on the basis of conflicting evidence, which increases the learning cost (Xie & Mack, 2024).

Perhaps the strongest reason to teach exception words early on during phonics instruction is that they enable students to progress from reading curated lists of regular example words to reading simple sentences and, eventually, naturalistic text. Given the high frequency of many exception words in language (see above), late teaching of these words may delay the point at which naturalistic reading is possible. However, although reading text is the obvious end goal of phonics instruction, exactly when to transition from reading isolated words to words in context is an area of debate. Both isolated and contextual practice have been shown to yield distinct benefits for reading development during the first years of elementary school—that is, well beyond the period of initial phonics instruction (for reviews, see Li & Wang, 2023; Nation & Castles, 2017). Whereas isolated reading draws learners’ attention to the orthographic details of words, thus supporting later rereading (Ehri & Roberts, 1979; Johnston, 2000; Landi et al., 2006) and spelling (Martin-Chang et al., 2017), context provides semantic and morphosyntactic cues that help children decode orthographically unfamiliar words and strengthen the links between orthography, phonology, and semantics (Archer & Bryant, 2001; Cunningham, 2006; Martin-Chang et al., 2007; Wang et al., 2011).

As a result, deciding when to teach phonics exceptions may require further research into what skills teachers should prioritize, and for how long. If the evidence calls for a longer period of isolated word practice in which students’ knowledge of spelling-sound mappings is firmly entrenched, then it may be preferable for teachers to delay the teaching of (most) exception words. If, instead, the evidence supports a quick transition to reading in context and the additional semantic scaffolding it provides, then it may be preferable for teachers to introduce exception words earlier on.

### Interim summary and future directions

In sum, research on reading and learning suggests that decisions on which exception words to teach, and when to introduce them, are influenced both by the linguistic properties of exception words (such as their frequency) and by the target skills that are emphasized during early literacy education. Regarding the latter, the development of robust decoding skills may benefit from a delayed introduction of exception words, whereas a quick transition to naturalistic text reading may require earlier teaching of these words. Future work could use experimental and computational methods to determine an optimal trade-off between these competing factors. The results could inform comparisons of the efficacy with which existing phonics programs use exception words and aid the development of optimized exception sets and schedules for teaching them.

## Moving Forward: The Road Toward Optimized Phonics Content

**Fig. 6.**
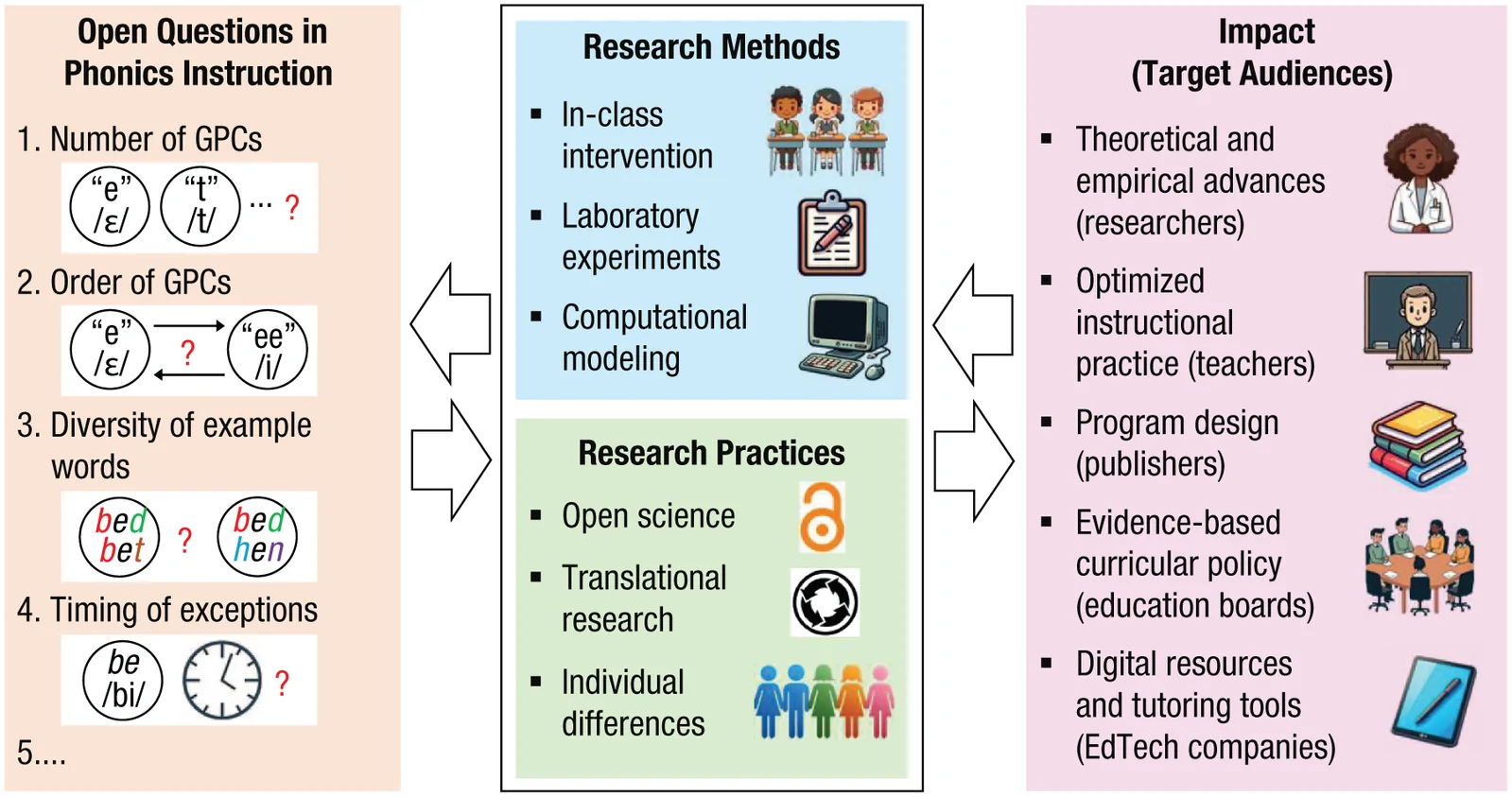
Road map for future research on the content of phonics instruction. GPCs = grapheme-phoneme correspondences; EdTech = educational technology.

We have illustrated that existing work from the reading sciences and allied research fields can inform key aspects of phonics instruction, but that such research also raises important questions for follow-up. In Figure 6, we summarize our discussion and provide a road map for future research on the content of phonics instruction. However, as we further address below, many of the principles we outline can also be applied to other areas of reading instruction. The three columns of the diagram are related by bidirectional arrows to highlight that successful phonics research relies on a continuous cycle of mutual feedback shaped by the questions that are being investigated, the methods and research practices used to examine them, and the impact of the research for diverse target audiences.

### Open questions in phonics instruction

Starting on the left of Figure 6, the first column summarizes key questions about the content of phonics instruction that we have addressed in this article, which are informed by existing research but also remain targets for future study and optimization. This list could be extended with further aspects, some of which we have mentioned; examples include the microsequencing of teaching and the debate about blocked versus interleaved learning. Other topics could be added—for instance, best practices in the review of phonics knowledge (see Jones & Reutzel, 2012; Jones et al., 2013). Notably, many of these factors are interrelated and mutually constrain each other: For instance, if more GPCs are taught, then fewer words may need to be introduced as exceptions from those rules. Similarly, as we have noted above, the diversity of example words may have different effects on learning depending on whether GPCs are taught in blocked or interleaved fashion. As a result, future research needs to study the aspects discussed here as interlocking pieces of a puzzle that together inform effective phonics instruction.

Even though our focus in this article has been on English, the broader principles we have discussed—for instance, the factors influencing GPC selection, the characteristics of effective example words, and the graded nature of exceptions—will apply to many alphabetical writing systems. However, English remains, to some degree, an outlier among the languages of the world, especially with respect to its orthographic opacity (Share, 2008). At a more fine-grained level, different strategies may therefore be needed to optimize phonics content in varying languages. For example, in orthographically transparent languages such as Serbo-Croatian (Latin and Cyrillic scripts) or Korean (Hangul script), which display nearly perfect one-to-one mappings between spelling and sound (Cho, 2009; Turvey et al., 1984), the selection of GPCs is straightforward, so the focus of future work may be on identifying optimal example words to illustrate these GPCs and foster generalization. In contrast, a language such as Hebrew, in which typically only consonants but no vowels are written (Share & Levin, 1999), poses additional instructional challenges, such as teaching children to infer the implied vowels on the basis of the meaning of words in context.

### Research methods

The top middle of Figure 6 summarizes the central methods used in research on phonics instruction, each of which has its distinct advantages and limitations. In-class intervention studies (also called *randomized controlled trials*, or RCTs) provide ecologically valid, holistic, and potentially longitudinal insights about the efficacy of teaching approaches, but they are resource-intensive and make it difficult to isolate the effects of specific instructional components in an efficient manner. Lab experiments, on the other hand, can serve to address targeted questions in a controlled, small-scale setting—for example, by training participants on a curated set of nonwords (i.e., newly invented words that consist of familiar letters; see, e.g., Nation et al., 2007), novel GPCs based on letters from an unfamiliar language (e.g., Law et al., 2018), or words spelled in an artificially created orthography (Taylor et al., 2011), either with or without explicit instruction and feedback. These studies allow researchers to manipulate individual experimental variables, and they are resource-efficient, faster to conduct, and easier to replicate, but the generalizability of their findings may be limited by the use of constrained tasks and test settings that have low ecological validity.

Of particular promise may be approaches that occupy the middle ground between long-term interventions and single-session lab experiments—for instance, by training participants over multiple days (or even weeks) and using a variety of experimental tasks to assess their reading performance. Studies that display these characteristics have been conducted both with adult participants in the lab (e.g., Rastle et al., 2021; Taylor et al., 2017) and with children in classroom settings (or classroom-adjacent settings; e.g., Apfelbaum et al., 2013; Roembke et al., 2020; see also Vazeux et al., 2020, for a related approach to studying phonemic awareness). The latter investigations have been described as field tests of learning principles in real-world educational settings (McMurray et al., 2019; see also Roberts, 2021, for a related discussion of the value of multiweek intervention studies with small student groups). We view work of this type to be particularly valuable in bridging the gap between naturalistic classroom-based trials and targeted lab experiments, allowing for the evaluation of the scalability of a particular theory from a highly constrained laboratory context to a more realistic one without fully committing to testing an entire program in several classrooms.

Finally, computational models can be used to simulate learning and teaching processes in a time-efficient and cost-effective way, providing formally precise descriptions of the multifactorial relationships that can exist between spelling and sound. This is true both in terms of proof-of-concept simulations related to simple, highly controlled lab experiments as well as more complex simulations that mimic the learning of thousands of words and are intended to better approximate the richness and complexities associated with learning to read in a real language (see Kello & Plaut, 2003, on fundamentalist versus realist models of reading for related discussion). By extending classic computational accounts of skilled reading (e.g., Plaut et al., 1996), recent work has, for instance, used neural networks to simulate the potential impact of different sets of example words (Cooper Borkenhagen et al., 2025) or entire phonics programs (Armstrong et al., 2024) on reading performance, and to generate improved word lists and GPC teaching progressions (Potier Watkins et al., 2019). However, the validity of simulated results depends on the assumed linkage between human and artificial cognition, which, although frequently substantiated (e.g., Chang et al., 2020; McClelland & Rogers, 2003; Plaut et al., 1996), is not guaranteed (Ludwig et al., 2025; Marcus, 2018) and must be corroborated through coordinated empirical investigations in the lab and in the classroom.

Notably, the aforementioned methods, as well as the research goals and approaches they are typically associated with, are often siloed within the different disciplines that, as we noted at the outset of this article, make up the reading sciences. For example, whereas educational psychologists typically rely on large-scale intervention studies to measure reading performance in real-life classroom settings, cognitive scientists tend to use smaller-scale lab experiments or computational models to study fundamental aspects of reading. Critically, these different research strands, which are frequently pursued at different university departments or colleges, are not always sufficiently connected, and mutual knowledge of, and training in, the methods used by the other disciplines can be limited. To a significant extent, therefore, the reading sciences constitute a multidisciplinary ensemble of heterogeneous, partly interconnected fields, but they have arguably not achieved true *interdisciplinarity*, which involves “analyz[ing], synthesiz[ing] and harmoniz[ing] links between disciplines into a coordinated and coherent whole,” or *transdisciplinarity*, which integrates fields in a way that “transcends their traditional boundaries” (Choi & Pak, 2006, p. 351).

Ample opportunities remain for future research on reading instruction to harness the symbiotic potential of combining multiple methods. For example, lab experiments and computational simulations can be used to narrow down the hypothesis space and pretest assumptions that can then inform the design of intervention studies, thus preventing the suboptimal use of time and resources. Intervention studies in classrooms, in turn, are crucial for determining whether theories developed at the level of basic science in the lab can scale up to, and meaningfully predict, learning outcomes in the classroom. Such work can also help guide decisions regarding what, in theory, could be taught, versus what can actually be taught in practice. For instance, as discussed above, basic science has revealed hundreds of GPCs that could be taught, but fewer than 100 are taught in each of the programs we examined, frequently because the programs opt to teach only the most frequent regularities rather than subordinate regularities that reflect rare or alternative ways of pronouncing the same letters. Recommendations from basic science must therefore be constrained by the everyday realities of classroom instruction, integrating the expertise and practical experience of teachers and other experts in education into the research process, as further addressed below.

### Research practices

The bottom middle of Figure 6 shows research practices that, in our view, are crucial for bridging the gap separating the reading sciences from their application to classroom practice (Seidenberg et al., 2020; Solari et al., 2020). First, effective phonics research should draw on the principles of *open science*: In order to crack open the “black box” of existing phonics programs, evaluate their design choices, and develop optimized sets of teaching content, researchers must transparently report their methods, provide reproducible results, and communicate their findings in clear and accessible ways. Open science also includes free sharing of research resources, including, for instance, publicly available databases of picture books (Dawson et al., 2021; Green et al., 2024) and children’s books (Korochkina et al., 2024) that can be used to select age-relevant stimuli for experiments or to test the performance of computational models. In the case of commercial phonics programs, increased emphasis should be placed on outlining the scientific basis for key decisions made in determining and structuring the content. This will allow researchers and educators to evaluate whether a program adheres to evidence-based practices known to promote learning.

Second, research on phonics should be *translational*, aiming to connect basic research on reading, learning, and language development with its applications in real educational settings. Such knowledge transfer must be inherently bidirectional, consisting of a mutual feedback cycle between scientific discovery and the practical expertise of teachers and other educational stakeholders. In our present discussion, for instance, we have utilized prior research to derive practical considerations and potential improvements for selecting effective phonics content in relation to the real-world requirements of classroom teaching (e.g., time constraints). Another area in which teachers’ expertise may crucially inform future translational research, and which could form the basis for another critical review in its own right, concerns the question of *how* this content is best communicated—that is, what teaching methods, strategies, and materials would be part of optimal implementations. For example, when teaching exception words, instructors not only need to decide what words to teach and when to introduce them, but also whether to teach students explicit strategies for reading exception words, how to prompt their use of such strategies, and whether to practice exception words through reading, spelling, or both (see Colenbrander, Kohnen, et al., 2022, on the latter question).

Third, although our focus in this article has been on phonics instruction delivered to whole student groups, research on effective phonics content should also take into account individual differences in learners’ linguistic abilities, such as vocabulary size and phonological-processing skills (Steacy et al., 2017; Wagner & Torgesen, 1987), and general cognitive skills, such as statistical-learning ability (Arciuli & Simpson, 2012). Individual students may, for example, benefit from varying amounts of phonics content, different teaching rates, or varying teaching orders (e.g., in the relative sequencing of GPCs and exception words), thus informing the nature of interventions designed for struggling readers (Bradley & Noell, 2018; Dilgard et al., 2022).

### Impact and target audiences

Finally, the right side of Figure 6 summarizes the potential impact of the present research directions for different target audiences. First, a better understanding of the factors that underlie effective phonics instruction can inform researchers’ work in diverse fields, including reading acquisition and literacy education, but also broader work on instructional design, learning and memory, and language impairments, among other areas. Insights gained, for instance, about the respective contributions of rules (GPCs), example words, and exception words, or about an effective ordering of these components, may be transferable to learning and teaching in a variety of other domains. Second, research on phonics instruction provides evidence-based considerations and guidance that can inform teachers’ decision-making in the phonics classroom. Specifically, this involves an increased awareness of what factors influence the success of particular aspects of teaching and, potentially, the selection of optimized teaching content.

As a third area of impact, this work invites educational publishers to reflect on the varying choices made during the design of phonics programs, and the reasons for which they are made, thus supporting the refinement and transparent evaluation of the programs and related teaching materials (e.g., workbooks, decodable readers). Fourth, it provides guidance for education boards at the local, provincial, state, and national level in implementing curricular policy, including the creation of curriculum standards, the evaluation of instructional materials, and the administration of standardized learning assessments. Fifth, insights about phonics instruction can be leveraged by educational technology companies to refine tools for self-directed learning within and outside the classroom, including adaptive learning software, digital tutors, and educational games (for discussion, see McTigue et al., 2020; Nicholas et al., 2017; Parry et al., 2024).

Finally, although we have illustrated the above road map in the context of phonics instruction, the general principles that guide it—in particular, a deeper integration of theories and methods from multiple reading sciences with the needs and expertise of diverse stakeholders in education—are broadly applicable to many other aspects of instruction that cover children’s long journey toward skilled reading. For example, past psycholinguistic research has indicated that teaching children to decompose words into their morphological components benefits reading, but the complex morphological curricula developed on this basis were found to be “very challenging to deliver” by the school workforce in an initial trial (Colenbrander, Parsons, et al., 2022, p. 327). Such pitfalls, in which findings from basic science are used to propose instructional regimes that are not feasible in practice, could be avoided by harnessing the symbiotic potential of combining lab-based studies with in-class trials and incorporating practitioners’ expertise early in the research process. The latter research practices could also help optimize instruction in other key skills that enable successful reading comprehension, including vocabulary knowledge, sentence and discourse processing skills, and the use of explicit strategies such as text summarization and prediction (see Castles et al., 2018).

Applying our road map to multiple aspects of reading instruction, starting with phonics and extending to other core components taught over many years in the classroom, could have enormous societal benefits. For example, in the United States, it has been estimated that obtaining minimum literacy proficiency for all adults could generate an additional US$2.2 trillion, or 10% of GDP in general income (Rothwell, 2020; see also Crawford et al., 2025, for a global perspective). Consequently, it is hard to overstate how much there is to be gained from improving current educational and research practices through the approach we have illustrated here.

## Conclusion

In this article, we have addressed how state-of-the-art research on reading, learning, and memory can inform practical issues related to phonics instruction, thus narrowing the gap that separates the reading sciences from their application to early literacy education. In particular, existing research provides key findings and considerations regarding the content of effective phonics instruction: for instance, what and how many GPCs to teach, in what order to teach them, what example words to use to illustrate GPCs, and when to introduce exceptions from spelling-sound regularities. This broad evidence base can be used to scrutinize and enhance current instructional practices in phonics classrooms, thus further showcasing the benefits of phonics and encouraging more school boards around the world to adopt a phonics-based approach to early literacy education. One reviewer of this work lamented that the impact of optimizing phonics instruction may be limited by the challenge of getting schools to use any form of phonics instruction, and we agree, but we would also highlight that what may look like incremental improvements in instructional effectiveness may have a very large impact, especially when applied at population scale.

In addition, we have outlined a road map for how future work on phonics instruction—which would largely build on existing, well-established methods from the reading sciences and thus form a tangible extension of prior work—could further inform these applied goals. Specifically, a combination of experimental and computational approaches could serve to compare the efficacy of varying sets of phonics content currently taught in the classroom and to develop new, optimized content and teaching schedules. Apart from its implications for basic science, the resulting evidence could be used by teachers to support their instructional practice, by educational publishers to enhance the design of phonics programs, by education boards to inform curricular policy, and by educational technology companies to develop and refine personalized educational technology. Extending beyond phonics, the interdisciplinary, translational research practices we have highlighted could also be applied to other core components of reading education, thus providing optimized, evidence-based instructional guidance to foster, for instance, children’s morphological, sentence, and discourse processing skills or their use of explicit text comprehension strategies.

In our view, therefore, the existing work and future directions discussed here put the reading sciences in a strong position to answer the skeptics’ call and “speak to what to teach, when, how, and for whom at a level that is useful for teachers” (Seidenberg et al., 2020, p. S121).

## Transparency

*Action Editor:* Moira R. Dillon

*Editor:* Arturo E. Hernandez

## Notes

1. All phonemic transcriptions in this article are based on English as spoken in Toronto, a variant of North American English; however, our conclusions are not affected by the choice of dialect.

2. Rognan, Champagne, et al.’s (2026) counts differ from those provided by Solity (2020) in an analysis of the same phonics programs. However, given that the full data and details of the analysis are not available for Solity’s study, we report Rognan, Champagne, et al.’s findings here because their data and methods are openly accessible and reproducible.

3. The GPC counts reported here encompass a few instances of rules (8%–19%, depending on the program) that map a single grapheme to multiple phonemes (e.g., “qu” – /kw/ or “tion” – /ʃən/). Even though these are not GPCs in the strict sense, defined as mappings from single graphemes to single phonemes (e.g., Coltheart et al., 2001, p. 212), we include them here because they are treated as spelling-sound rules in the programs. Excluding these items from our analyses does not alter any of our key claims.

4. Blocked learning and interleaved learning, as discussed here, are related to but nevertheless distinct from the contrast between massed and distributed practice (Mawson & Kang, 2025). The former concepts refer to the sequencing of distinct content elements (in this case, different GPCs) when they are first taught, whereas the latter terms relate to how the same content element (e.g., a single GPC) is repeatedly practiced over time.

5. In some cases, programs provide intervals of multiple weeks for the introduction of particular exceptions. In our diagram, we depict this by smoothly increasing the exception count during these intervals.

6. However, among the three programs examined by Rognan, Champagne, et al. (2026), Letters and Sounds is the only one in which a significant proportion of exception words (46%) are subsequently reused as examples of a GPC. Few instances of this type are attested in Jolly Phonics (7%) and Read Write Inc. (6%).
